# Disentangling Microbial Mediators of Malnutrition: Modeling Environmental Enteric Dysfunction

**DOI:** 10.1016/j.jcmgh.2018.12.006

**Published:** 2019-01-07

**Authors:** Luther A. Bartelt, David T. Bolick, Richard L. Guerrant

**Affiliations:** 1 Division of Infectious Diseases, Department of Medicine, University of North Carolina at Chapel Hill, Chapel Hill, North Carolina; 2 Center for Gastrointestinal Biology and Disease, Department of Medicine, University of North Carolina at Chapel Hill, Chapel Hill, North Carolina; 3 Center for Global Health, Division of Infectious Diseases and International Health, Department of Medicine, University of Virginia, Charlottesville, Virginia

**Keywords:** Environmental Enteropathy, Environmental Enteric Dysfunction, Malnutrition, Intestinal Barrier, Enteropathogen, AGP, α1-acid glycoprotein, A1AT, α1-antitrypsin, BG, Bacteroidiales mix, CRP, C-reactive protein, EAEC, enteroaggregative *Escherichia coli*, EE, environmental enteropathy, EED, environmental enteric dysfunction, ETEC, enterotoxigenic *Escherichia coli*, FC, fecal calprotectin, fMPO, fecal myeloperoxidase, HAZdrop, decrease in height for age z-score, IEC, intestinal epithelial cell, IEL, intraepithelial lymphocyte, IFN, interferon, IGF-1, insulin-like growth factor 1, IL, interleukin, LCN-2, fecal lipocalin-2, L:M, lactulose:mannitol, LPS, lipopolysaccharide, PM, protein malnutrition, TJ, tight-junction, TLR, Toll-like receptor, TMA, trimethylamine, TMAO, trimethylamine oxide, ZD, zinc-deficient

## Abstract

Environmental enteric dysfunction (EED) (also referred to as environmental enteropathy) is a subclinical chronic intestinal disorder that is an emerging contributor to early childhood malnutrition. EED is common in resource-limited settings, and is postulated to consist of small intestinal injury, dysfunctional nutrient absorption, and chronic inflammation that results in impaired early child growth attainment. Although there is emerging interest in the hypothetical potential for chemical toxins in the environmental exposome to contribute to EED, the propensity of published data, and hence the focus of this review, implicates a critical role of environmental microbes. Early childhood malnutrition and EED are most prevalent in resource-limited settings where food is limited, and inadequate access to clean water and sanitation results in frequent gastrointestinal pathogen exposures. Even as overt diarrhea rates in these settings decline, silent enteric infections and faltering growth persist. Furthermore, beyond restricted physical growth, EED and/or enteric pathogens also associate with impaired oral vaccine responses, impaired cognitive development, and may even accelerate metabolic syndrome and its cardiovascular consequences. As these potentially costly long-term consequences of early childhood enteric infections increasingly are appreciated, novel therapeutic strategies that reverse damage resulting from nutritional deficiencies and microbial insults in the developing small intestine are needed. Given the inherent limitations in investigating how specific intestinal pathogens directly injure the small intestine in children, animal models provide an affordable and controlled opportunity to elucidate causal sequelae of specific enteric infections, to differentiate consequences of defined nutrient deprivation alone from co-incident enteropathogen insults, and to correlate the resulting gut pathologies with their functional impact during vulnerable early life windows.

**Summary**

Environmental enteric dysfunction is a common but poorly understood disorder associated with early childhood malnutrition. In this review, we appraise the application of murine models to advance understanding of how specific intestinal microbes contribute to the development of environmental enteric dysfunction.

Environmental enteric dysfunction (EED; also known as environmental enteropathy [EE]) is a subclinical chronic intestinal disorder that is an emerging contributor to early childhood malnutrition.^1^ EED is common in resource-limited settings, and is postulated to consist of small intestinal injury, dysfunctional nutrient absorption, and chronic inflammation that results in impaired early child growth attainment.^1^, ^2^, ^3^, ^4^, ^5^ Although there is emerging interest in the hypothetical potential for chemical toxins in the environmental exposome to contribute to EED,^6^, ^7^, ^8^ the propensity of published data, and hence the focus of this review, implicates a critical role of environmental microbes. Early childhood malnutrition and EED are most prevalent in resource-limited settings where food is limited, and inadequate access to clean water and sanitation results in frequent gastrointestinal pathogen exposures.^9^, ^10^, ^11^ Even as overt diarrhea rates in these settings decline, “silent“ enteric infections and faltering growth persist.^12^, ^13^ Furthermore, beyond restricted physical growth (decrease in height for age z-score [HAZdrop]),^14^ EED and/or enteric pathogens also associate with impaired oral vaccine responses,^15^ impaired cognitive development (cognitive impairment hit),^16^ and may even accelerate metabolic syndrome and its cardiovascular consequences.^17^, ^18^ As these potentially costly long-term consequences of early childhood enteric infections increasingly are appreciated, novel therapeutic strategies that reverse damage resulting from nutritional deficiencies and microbial insults in the developing small intestine are needed. Given the inherent limitations in investigating how specific intestinal pathogens directly injure the small intestine in children, animal models provide an affordable and controlled opportunity to elucidate causal sequelae of specific enteric infections, to differentiate consequences of defined nutrient deprivation alone from co-incident enteropathogen insults, and to correlate the resulting gut pathologies with their functional impact during vulnerable early life windows.

## Definition of EED

Despite coordinated global efforts to improve the nutritional status of malnourished children, the protein-rich complementary foods combined with vitamin A and zinc supplementation, breastfeeding promotion, and prenatal micronutrient supplementation are predicted to decrease global linear growth restriction (stunting) by only a third.^19^ The lackluster outcomes of these interventions have led to a resurgence in epidemiologic and pathogenesis research focused on what factors drive and sustain malnutrition.

The concept that childhood malnutrition is caused not only by nutrient depravation, but is at least in part a result of underlying intestinal dysfunction, has been evolving for more than 50 years. In the 1960s, small intestinal villous flattening^20^ was first observed in children with kwashiorkor-type (protein energy) malnutrition.^1^ A decade later, similar structural pathologies were observed in North American Peace Corps volunteers during their service, and these abnormalities recovered upon repatriation.^21^ Subsequently, it was recognized that many children across geographically widespread resource-limited settings also show markers of intestinal inflammation (eg, fecal neopterin and fecal myeloperoxidase [fMPO]).^22^ These inflammatory markers that are otherwise uncommon in children in resource-abundant settings are associated with growth shortfalls as well as small intestinal villus blunting, and/or increased intestinal permeability. More recently, stunting in these children also was associated with increased serum inflammatory markers (eg, C-reactive protein [CRP] and α1-acid glycoprotein [AGP]).^23^, ^24^, ^25^ Several other alterations in the intestinal microbiome, other biomarkers of intestinal inflammation, and metabolic perturbations have since been associated with poor growth. The term *EED* is used to describe these clusters of findings suggestive of impaired gut function with clear geographic associations. Although a consensus definition of EE/EED is still in progress,^1^ our use of EE throughout this review therefore is meant to include both pathophysiology^2^ and pathological function that is also referred to as EED.^3^

## EED Knowledge Gaps

Unlike other small intestinal inflammatory disorders, such as gluten-sensitive enteropathy, no single-culprit environmental factor has been identified as a cause of EED. Although there is emerging interest in the hypothetical potential for chemical toxins in the environmental exposome to contribute to EED,^6^, ^7^, ^8^ the propensity of published data, and hence the focus of this review, implicates a critical role of environmental microbes. In addition to diminished nutrient availability, precedent and recurrent episodes of infectious diarrhea also associate with childhood growth restriction.^6^, ^26^ Even as diarrhea incidence and severity has decreased since the 1960s, approximately 500,000 global childhood deaths annually remain attributed to enteric infections.^14^ Likewise, the rates of stunting have been relatively stagnant. Emerging evidence from global studies in birth cohorts now show that cumulative, silent enteropathogen exposures, even in the absence of diarrhea, are associated with childhood stunting^10^, ^11^ and/or altered intestinal permeability.^10^, ^27^ Specific pathogens (such as norovirus, *Campylobacter* species, heat-labile toxin (LT)-enterotoxigenic *Escherichia coli* (ETEC), *Shigella*, *Giardia*, and *Cryptosporidium* among others) are associated independently with growth restriction, however, no single microbe has been identified as solely responsible for EED. Current epidemiologic findings have suggested that EED results from the convergence of nutrient deficiencies and multiple co-pathogens, potentially operating through distinct pathways. How quantitative pathogen-attributable burden influences growth restriction severity and variability across geographic sites and ages^28^, ^29^ requires further study.^28^ These analyses will help to clarify whether and to what extent specific pathogens likely operate through EED or EED-like pathways to promote malnutrition.

The outcomes of recent trials support the need for a deeper understanding of how subclinical intestinal pathogen exposures may contribute to intestinal dysfunction. Rejuvenating intestinal epithelial cells through nutrient-based remedies may be only transiently beneficial.^30^ Micronutrient supplementation^31^ or just zinc,^32^ can partially improve permeability (as measured by lactulose:mannitol [L:M] ratios), but not to normal/healthy values. Alanyl-glutamine, a fuel for epithelial cells, also improves permeability as well as child weight, but does not promote linear growth.^33^ One explanation for this limited benefit could be ongoing damage from intestinal inflammation. Targeting intestinal inflammation with mesalamine, however, did not promote growth in children with severe acute malnutrition, despite evidence of diminished systemic inflammation.^34^ Ongoing insults from intestinal pathogens could limit either nutrient- or anti-inflammatory–based therapies. Knowledge gaps remain, however, in our understanding of which microbes are most relevant for EED.^35^ Antibacterial therapy also has led to mixed results. Either amoxicillin or cefdinir decreased mortality and accelerated recovery among children with severe acute malnutrition,^36^ however, in a separate study amoxicillin had no apparent benefit in children with less severe malnutrition.^37^ The luminal agent rifaximin did not improve L:M ratios 3 weeks after treatment.^38^ Targeting intestinal parasites, such as *Giardia*, also had little effect on stunting or intestinal permeability, and *Giardia* re-infection was rapid.^39^ Complicating these findings, multiple and often nonprescription courses of broadly active antibiotics are common in many malnourished children.^40^ Despite potentially promoting weight gain, unsupervised antimicrobials do not appear to decrease stunting.^41^ Even when combined as a triple-therapy intervention of micronutrients plus zinc plus albendazole, neither linear growth attainment nor biomarkers of EED improved.^42^ Probiotic approaches have shown safety but have yet to establish efficacy for promoting growth or reducing diarrhea in these children.^43^ Finally, despite clear associations between environmental soil and fecal contamination and malnutrition, interventions to improve water, sanitation, and hygiene (without improved public sewage and water systems^44^) have resulted in only a small, if any, ability to reduce stunting at the population level.^45^ Thus, a better understanding of how hosts and specific microbes adapt to select nutritional deficiencies and how these microbial interactions shape host intestinal function, inflammation, metabolism, and growth is needed.

## Translating EED and EED-Like Conditions in Mice

### Mucosal and Functional Correlates of EED in Children

Emerging findings from comprehensive longitudinal analyses of children with poor growth are formulating clearer, if not highly complex and multifaceted interactions, between functional impairments, small intestinal pathology, microbial influences, biomarkers, and metabolic profile perturbations associated with EED (Supplementary Table 1). Characteristic upper small intestinal histopathologic features of EED overlap with, but also are distinct from, other chronic small intestinal enteropathies such as gluten-sensitive enteropathy and inflammatory bowel diseases. EED can show villous atrophy often with crypt hyperplasia,^20^, ^46^ epithelial barrier disruption^47^ corresponding to altered small intestinal permeability, 35, 38, 48, 49, 50 and aberrations in tight-junction (TJ) proteins. Subepithelial features include lymphocyte infiltration into the lamina propria^20^, ^46^, ^51^, ^52^ comprising B cells and activated T cells,^46^ local immune dysregulation,^53^, ^54^ and increased interferon γ (IFNγ) with relatively reduced interleukin (IL)10.^49^

Given that direct examination of upper small intestinal pathology in children with EED is seldom performed, functional and trackable markers relating impaired growth with intestinal barrier function, mucosal inflammation, and systemic inflammation are used in birth cohort studies. Increased intestinal permeability frequently is used as a marker of intestinal dysfunction. Intestinal permeability often is measured comparing absorption of the sugar alcohol mannitol with the otherwise poorly absorbed disaccharide lactulose.^55^ An increased L:M ratio is indicative of a leak of lactulose across the epithelial barrier. L:M ratios, however, can be cumbersome.^56^ Although altered L:M ratios in the first 2 years of life can persist even until adulthood,^57^ they are subject to dynamic and geographic variations that are both sex- and age-dependent.^27^ The L:M ratio also is just one measure of small-molecule permeability rather than a direct assessment of TJ proteins (ie, claudin-4) or barrier modulators (ie, zonulin). Increased serum intestinal fatty-acid binding protein is a more direct marker of intestinal injury that correlates with increased permeability. Intestinal fatty-acid binding protein also is increased together with activation of intestinal myeloid, B and T cells,^58^ and specifically fMPO.^24^, ^59^, ^60^ Impaired permeability can facilitate microbial translocation inferred by increases in circulating anti-lipopolysaccharide (LPS)^61^ or antiflagellin antibodies.^62^ This increased microbial translocation can promote systemic inflammation as measured by CRP, AGP, and kynurenine as potential markers of endogenous immune-mediated tryptophan metabolism.^24^, ^63^, ^64^, ^65^

Mucosal inflammatory markers of EED correlate with growth restriction and enteropathogen exposures. Total enteropathogen exposure burden is associated with increased fMPO (as well as fecal lipocalin-2 [LCN-2] and calprotectin [FC]). fMPO is associated most strongly with prototypically proinflammatory pathogens (such as *Shigella*/enteroinvasive *E coli* (EIEC) and *Campylobacter* species^66^), but not with other pathogens that are associated with both growth impairment and intestinal permeability (ie, atypical enteropathogenic *E coli* (aEPEC), *Cryptosporidium*,^29^ and *Giardia*^67^, ^68^).^69^ Although enteroaggregative *Escherichia coli* (EAEC), especially with fMPO, is associated with growth impairment, L:M ratios were not altered substantially.^70^ In contrast, altered L:M ratios were associated with *Giardia* exposure, but *Giardia* was associated with decreased fMPO.^67^, ^69^ Similar to the heterogeneity observed in permeability assays,^27^ fMPO shows substantial within-child variability, making individual interpretation difficult.^71^

Emerging metabolomics studies have shown that stunted children show derangements in protein metabolism such as increased endogenous conversion of tryptophan to kynurenine,^24^, ^72^ as well as increased exogenous breakdown of tryptophan and other aromatic amino acids (phenylalanine and tyrosine) by intestinal microbes. Severe villus blunting, however, may decrease systemic detection of microbial-mediated metabolites of proteolysis.^73^ Muscle breakdown products, such as creatinine,^73^, ^74^ as well as microbial conversion of choline to trimethylamine (TMA),^74^ also are increased in malnourished children.

Longitudinal studies across various geographic settings have attempted to integrate these multiple functional, pathologic, microbial, inflammatory, and metabolic read-outs. Although ongoing analyses provide useful insights into complex interactions supportive of the EED paradigm, a defined clinical panel of EED diagnostics and precise cut-off values have yet to be determined. Furthermore, there remains much uncertainty regarding which components are sufficient, or necessary, for resulting growth impairment. For example, in Pakistan, both gut and systemic inflammatory markers correlated with linear growth restriction and diminished insulin-like growth factor 1 (IGF-1). However, the gut and systemic markers correlated only weakly with one another.^25^ For some biomarkers, such as serum amyloid A, diminished levels correlated with stunting at enrollment, but increased levels at later time points are predictive of poor linear growth.^24^ Increases in inflammatory markers are not always coincident with altered permeability. Thus, just as no microbial agent sufficiently explains EED, isolated EED biomarkers are difficult to interpret. In addition, whether EED exists separate from repeated enteric pathogen exposures is unclear. Higher-resolution prospective epidemiologic studies to assess biomarker dynamics in children as they relate to specific pathogen infections and biological models therefore are attempting to disentangle how putative pathogen exposures may result in EED, and how resulting pathologies correspond to functional EED sequelae, biomarkers, and interventions during early life.

### Models of EED and EED-Like Conditions in Mice

**Figure 1:**
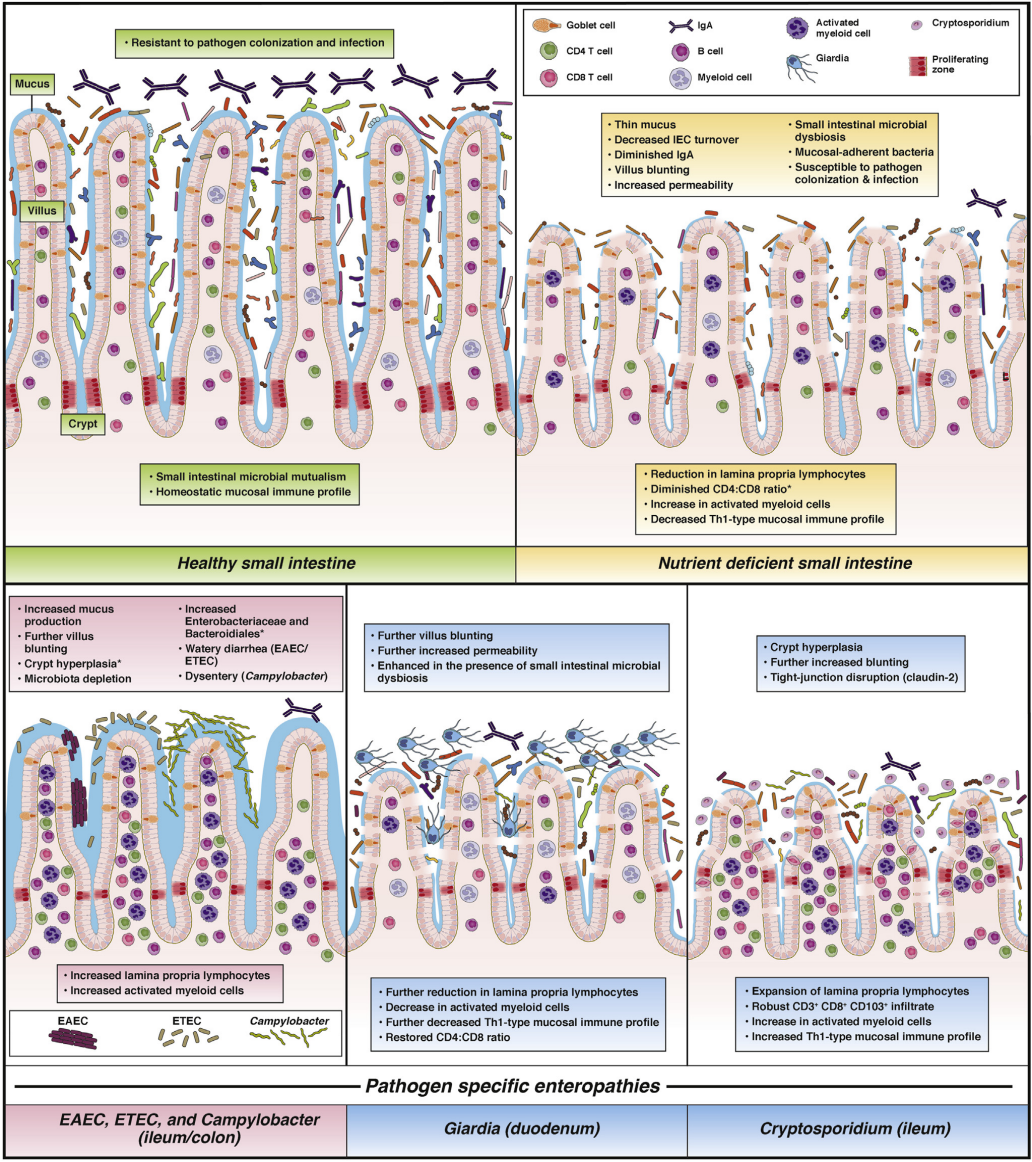
**Healthy small intestine (*green* boxes) contrasted with intestinal pathologies observed in murine models of nutritional deficiency alone (*yellowboxes*) compared with specific pathogen infection during either zinc deficiency (*pink boxes*) or protein deficiency (*blue boxes*).** *The described features are observed in models of protein deficiency. A diminished CD4:CD8 ratio is seen during both protein and zinc deficiency. Future applications of these models can help to address important knowledge gaps. How do genetic and epigenetic factors shape intestinal adaptations during select nutrient deficiencies? How do microbial communities and intestinal pathogens differentially adapt to select nutrient deficiencies in the small intestine, and what are the consequences of this altered microbial ecology on epithelial cell function, host nutrient availability, inflammation, and metabolism? What are the consequences of nutrient and microbial-dependent acute (episodic) and chronic (persistent) mucosal and systemic inflammation on intestinal function, susceptibility to infection, nutrient demand, metabolism, and host growth?

Murine models of malnutrition initially characterized the consequences of nutrient deficiency or caloric restriction without carefully controlling for the influence of environmental microbes, including pathogens, that are central to the paradigm of EED pathogenesis.^75^ Likewise, conventional pathogen challenge models in mice have been optimized for dramatic and acute phenotypes, rather than the more indolent subclinical features of EED. As in human studies, this gap in conventional models is especially true for juvenile or neonatal mice. New animal models are providing opportunities for more mechanistic understandings of EED and EED-like conditions (Figure 1). These models can assess intestinal pathologic features with EED biomarkers (Supplementary Table 1), as well as preclinical therapeutics (Table 1).

**Table 1:** Putative EED Therapies: Growth Outcomes in Pediatric Clinical Trials and Weaned Mouse Models

Therapeutic	Childhood EED	Weaned mouse model						
	Undefined pathogens	No pathogen	EAEC	ETEC	*Campylobacter*	*Giardia*	*Cryptosporidium*	References
Antimicrobial								
Nitazoxanide	Ongoing^a^	No difference	Prevents			No difference	No difference	120, 92, 94
Amixicile		Improves					Less severe	120
Luminal microbiota depletion								
Protein deficiency	No difference^36^, ^37^, ^38^	Improves	More severe	More severe	More severe	Prevents	No difference	91
Zinc deficiency		Restricts	More severe	Very severe	Very severe			101, 93, 97
Targeted nutrient therapy								
Alanyl-glutamine	WAZ not HAZ^b^^,^^33^	Improves^b^					Less severe	80, 94
Arginine							Less severe	123
Zinc	No difference^b^^,^^32^, ^42^	No difference		Restores				93
Mucosal immune modulation								
TLR9 agonist (CpG)		No difference					Less severe	82, 94
* S* Typhi vaccine		No difference					Partial recovery	82
Prior exposure							Prevents^c^	82
Probiotic								
*Lactobacillus* species^d^	No difference^43^	Improves						127

a ELICIT trial (NCT03268902) consisted of a combination of nitazoxanide + azithromycin + nicotinamide.

b Intervention either improved intestinal permeability, or prevented intestinal permeability deterioration.

c Only intervention in which reduced intestinal pathogen burden consistently accompanied growth benefit.

d *Lactobacillus rhamnosus* GG in children, *L plantarum*^WJL^ in mice.

### Epithelial Cell and Mucosal Defense Defects During Defined Nutrient Deficiency

Among the various models of malnutrition in animals,^76^, ^77^, ^78^ isocaloric protein malnutrition (PM) alone,^79^ or together with moderate fat deficiency,^80^, ^81^ is epidemiologically relevant to dietary deficiencies in children with EED.^11^ Findings across these models were reviewed recently by Attia et al.^75^ In summary, PM profoundly influences mucosal homeostasis, but it is not sufficient to account for all features of EED. Villus length decreases proportional to the severity of protein deficiency.^76^, ^81^, ^82^ Some of these defects can be restored with replenishment of alanyl-glutamine alone.^80^ Moderate protein deficiency (7% protein) is sufficient to alter intestinal permeability together with increases in gene expression for tight junctions such as claudin-2,^81^ and decreases in TJ protein 1 (which transcribes the protein zonula occludens 1).^81^ However, in separate studies of more severe protein deprivation (2% protein), immunofluorescence staining of TJ proteins showed reduced occludin but not zonula occludens 1, and no differences in claudin-2. Claudin-3 was found to be increased along with intestinal permeability in mice fed a moderate multinutrient-deficient diet.^80^ PM also has been shown to impair intestinal epithelial cell (IEC) proliferation as well as apoptosis, thus impairing an often unappreciated host defense of IEC turnover (potentially explaining the increased susceptibility and severity of mucosal epithelial infections such as cryptosporidiosis).^83^ Although villus blunting and altered permeability during PM are predicted to result in diminished absorptive capacity, impaired nutrient uptake has been difficult to prove. In contrast to PM, there is no apparent growth restriction in mice fed a severe zinc-deficient (ZD) diet for the few weeks studied.^79^ Despite clear in vitro effects on IEC membrane integrity during ZD, alterations of intestinal architecture or barrier disruption have not been reproduced consistently.

Disrupted architecture during early PM does not appear to be the consequence of impaired pro-proliferative signaling. In either protein-deprived mice or in vitro IEC starvation models there is up-regulation of proliferative intestinal cell kinase, Wnt/β-catenin, mammalian target of rapamycin, mitogen-activated protein kinase, and Akt (protein kinase B) pathways, and in response to protein deprivation^84^ apoptosis is reduced. A transient increase in Lgr5+ stem cells suggests compensatory responses in the stem cell niche during early time points of protein deficiency. Alterations in the growth hormone and IGF-1 axis resulting from PM could influence villus length, but have yet to be proven.^85^ However, altered IGF-1–receptor expression^86^ in apolipoprotein E knockout mice that have accentuated malnutrition and diminished catch-up growth upon refeeding^87^ suggest that genetic determinants influence intestinal adaptations to nutrient deprivation relevant to IGF-1 signaling.^88^

Undernutrition also results in changes in specialized epithelial cells. Mucus layer depth, goblet cell numbers, and mucus within goblet cells all are decreased, and goblet cell differentiation might be impaired.^75^ These changes correspond with findings of decreased mucin gene expression in children with EED,^58^ however, comprehensive characterization of disruptions in other epithelial cell types have not been examined thoroughly. Depriving Paneth cells of zinc limits their function, and although some Paneth cell antimicrobial peptides may be reduced in malnourished adults,^89^ these and other specialized epithelial types have not been scrutinized meticulously in children with EED.

Specific nutrient depletion alters baseline innate and adaptive mucosal immune responses. Fecal markers of epithelial cell (LCN-2) and neutrophil activation (MPO and LCN-2) are increased in mice with PM, but not zinc deficiency alone.^79^ Toll-like receptor (TLR)2 and TLR4, but not TLR9, expression is increased during PM,^90^ however, TLR4 signaling is not necessary for nutrient-dependent mucosal disruption.^81^ In contrast, and opposite to findings in EED, several mucosal proinflammatory cytokines, such as IL6 and IL12p40, were diminished in the upper small intestine during PM, whereas IL4 was increased.^91^ Similar to some malnourished children,^24^ mice fed a protein-deficient diet also show lower serum levels of the acute-phase reactant serum amyloid-A (L.A.B., unpublished data). Changes in IL17A have been variable across murine models (L.A.B., unpublished data). Alterations in cytokines during PM correspond with an overall depletion of T and B cells in the small intestinal lamina propria,^91^ but intraepithelial lymphocytes (IELs) may be increased.^81^ During either PM or ZD, CD4^+^ T cells are decreased disproportionally. Importantly, the diminished lamina propria lymphocyte numbers are fundamentally opposite of characteristic features of EED, further suggesting environmental or pathogen contributions beyond nutrient deprivation lead to EED.

Thus, dietary protein and, to a lesser extent, zinc, are critical mediators of mucosal homeostasis, particularly in regards to epithelial cell morphometry and function. Although PM is sufficient to enhance some proinflammatory mediators (such as fMPO), the chronic intestinal inflammation of EED likely requires additional specific microbial insults (Figure 1). It remains uncertain, however, whether and how specific microbes, including resident microbiota, differentially alter these host immune response adaptations during PM.

### Pathogen Susceptibility and Virulence During Nutrient Deficiency

Many of the enteropathogens detected in stunted children (eg, norovirus, *Campylobacter* species, *E coli* pathotypes, *Giardia*, and *Cryptosporidium*) localize and adhere to the small intestinal mucosa (Figure 1). Careful selection of pathogen strains originally isolated from human beings, and in the case of intestinal protozoa, the naturally infectious parasite stage, have proven important for optimizing infectivity and modeling features that overlap with EED. For example, infection with the EAEC042 strain that produces a repertoire of several virulence factors (*aap*, *virK*, *aaiC*, *aggR*) leads to greater growth impairment compared with a strain with more diminutive virulence gene expression.^92^ New models of ETEC have show shown differential effects of heat-labile toxin (LT) and heat-stable toxin (ST) on ETEC pathogenesis.^93^ Purified *Cryptosporidium parvum* oocysts and *Giardia lamblia* cysts are more infectious than conventionally used excysted *Cryptosporidium* sporozoites^94^ or axenized *Giardia* trophozoites.^91^, ^95^ These models have shown that PM promotes greater small intestinal burden of intestinal pathogens (eg, *G lamblia*, ∼0.5 log; *C parvum*, ∼3 logs; and EAEC042, ∼5 logs).^91^, ^94^ Despite this increased susceptibility to primary challenge, diminished IgA and IFNγ during PM, in the case of rotavirus^96^ and *C parvum*,^82^ protective host immune responses against rechallenge remain surprisingly robust. Similarly, these pathogens are of low or moderate recurrence in children. Pathogens that frequently persist in children, such as *Giardia* and EAEC, however, are capable of prolonged colonization in even healthy fully nourished mice if intestinal murine microbiota are depleted.^95^, ^97^ In the presence of resident microbiota, however, long-term persistence occurs only in mice with ongoing PM.

Specific nutrients can alter virulence expression of intestinal pathogens. Iron deficiency limits the pathogenicity of invasive *Salmonella* infections^98^ and alterations in iron metabolism among various *E coli* pathotypes differentially influences their proinflammatory potential in colitis models.^99^ Zinc, in contrast, diminishes EAEC042 virulence factor expression both directly and in vivo together with enhanced epithelial defense responses,^100^ whereas EAEC virulence gene expression is increased in ZD mice.^97^ Zinc deficiency also promotes ETEC virulence genes (*cfa1*, *cexE*, *sta2*, and *degP*).^93^ Thus, the complexity of microbe–host interactions increases as each adapts to the limited nutrient environment.

### Pathogen and Host Response–Mediated Epithelial Cell Injury

Direct pathogen-mediated epithelial cell damage is well documented in vitro, but whether these effects are mechanistically sufficient to result in EED has yet to be discerned. In vivo, only *Campylobacter jejuni* and ETEC consistently result in diarrhea, and only when in combination with ZD and microbiota depletion.^93^, ^101^ A cocktail of *E coli* and Bacteroidiales mix (BG),^81^ *Giardia*,^95^ EAEC, or *Cryptosporidium*^94^ can enhance villus blunting during PM, although only *Cryptosporidium* leads to significant crypt hyperplasia. In the case of *E coli* and Bacteroidiales^81^ and *Giardia* (L.A.B., unpublished data), intestinal permeability is impaired. Specific mechanisms accounting for increased barrier permeability have not been elucidated, but alterations in the TJ protein claudin-2 have been observed across several models.^81^, ^82^ Furthermore, although either *Giardia* or *Cryptosporidium* can lead to increased IEC apoptosis in vitro and in fully nourished animals, apoptosis as measured by anti–cleaved caspase 3 is not increased after infection during PM despite (and even potentially contributing to) greater parasite burdens.^83^, ^95^

These alterations in IEC homeostasis are most prominent in the intestinal region with the greatest pathogen burden and co-localize with mucosal cytokine responses that could further disrupt barrier function.^82^, ^91^, ^92^ Although fully nourished mice challenged with *C parvum* oocysts clear parasites with little evidence of a secondary immune response, even a low inoculum during PM coincides with the early release of chemokine chemokine ligand 5, increases in IFNγ levels, and recruitment of B and T cells into the lamina propria.^82^ In these studies, ongoing growth impairment persists even after *C parvum* clearance and may be a consequence of a robust influx of cytotoxic CD3^+^CD8^+^ T cells honed to the epithelial compartment.^82^ Similarly, although cytokine release from IELs in fully nourished mice repeatedly fed the BG cocktail remained muted, BG promoted tumor necrosis factor α, IFNγ, and, to a lesser extent, IL17A release in IELs of moderately malnourished mice.^81^ Either protein or zinc deficiency results in magnified myeloid cell responses to EAEC042, ETEC, and *Campylobacter* challenge,^91^, ^93^, ^97^, ^101^ consistent with increased fMPO in malnourished children infected with bacterial enteropathogen exposures.^69^ Thus, dysregulated proinflammatory cytokines and cytotoxic IELs in response to pathogens could enhance IEC permeability. More investigation is needed to determine the role of emerging innate mucosal defense axes in EED, such as the recognition that IL22 regulates claudin-2 to facilitate pathogen clearance.^102^

### Pathogen-Specific Immune Responses During Protein Malnutrition and EED

Mechanistic explanations for susceptibility to primary and multiple enteropathogen infections in malnourished children remain limited.^103^ Susceptibility appears to be pathogen-specific. Although cryptosporidiosis is worse in either malnourished children or mice,^82^ rotavirus diarrhea may be diminished during malnutrition in children,^104^ neonatal piglets,^77^ or mice.^96^, ^105^ In a cohort of Bangladeshi infants, children with fecal biomarkers of EED (including fMPO) showed impaired immunogenicity to oral rotavirus and polio vaccines, but immunogenicity to parenteral vaccines (such as tetanus) was preserved.^15^ Similarly, the severity of murine norovirus (strain MNV-1 isolate CW3) infection was amplified and memory responses to rechallenge were blunted and ineffective in mice with PM.^106^ Diet-dependent changes in resident microbiota during PM may account for at least some of the increased susceptibility to intestinal pathogens. Compared with fecal microbes from healthy controls, gnotobiotic piglets conventionalized with fecal microbes from children with increased fecal EED biomarkers showed diminished rotavirus-specific IFNγ-producing T cells (mucosal, blood, and spleen), however, neither rotavirus-specific IgA nor IgG neutralizing antibodies in serum were preserved.^107^

Although most studies in children and mice have focused on responses to primary infection, longitudinal rechallenge and vaccine studies during PM in mice have shown that despite ongoing protein deficiency, adaptive immune responses are at least partially preserved, and can remain pathogen-specific and protective. For example, although a calorie-restricted diet in gnotobiotic piglets conventionalized with infant microbiota impairs innate immune responses (natural killer, plasmacytoid dendritic cells, and CD103^+^ cells) and reduces serum IL12p40 in response to primary human rotavirus challenge,^78^ rotavirus vaccine efficacy in mice with PM is preserved despite diminished fecal IgA.^96^ Similarly, Th1-type proinflammatory cytokines appear globally diminished in mucosal compartments, mesenteric lymph nodes, and spleens of mice with PM,^82^, ^91^ yet select mucosal pathogen exposures can lead to sustained, and in some cases may at least partially re-establish, protective immune responses. IL17A responses to an attenuated oral *Salmonella* Typhi vaccine were preserved if not overexuberant during PM.^108^ Even remote mucosal exposure to this *Salmonella* Typhi vaccine vector devoid of *Cryptosporidium* antigens, or the TLR9 agonist CpG oligonucleotides, led to partial attenuation of severity of weight loss after *C parvum* challenge despite ongoing PM.^82^ Furthermore, mice with PM show surprisingly robust protection against both the severity and duration of *C parvum* shedding after homologous rechallenge.^82^ Concurrent studies with *G lamblia*, however, highlight that remodeling of host immune responses during PM is pathogen-specific. Although *Cryptosporidium* leads to partial re-establishment of Th1-type immune responses,^82^ *Giardia* magnifies an opposing diminished Th1-type profile, despite overlapping features of epithelial cell injury. These effects of *Giardia* on host responses during PM are sufficient to alter myeloid activation in response to EAEC042 (FC and fMPO).^91^ In this context, *Giardia* also has been found to be associated with diminished levels of serum CRP in children^109^ and may be uniquely anti-inflammatory among EED-associated pathogens.^69^

### Dysfunctional Resident Microbiota: Dysbiosis

Environmental and dietary factors increasingly are recognized to have a profound influence on composition and function of intestinal resident microbiota during early childhood.^110^ Although there is wide geographic variation in the 16S ribosomal RNA genomic composition of the microbiome of malnourished children,^111^, ^112^ a longitudinal feature of microbiome development in malnourished children is a persistent perinatal or immature profile.^113^ In weaned murine models, PM, but not ZD, results in a lag in microbiota maturation.^79^ Both moderate and severe PM regionally reshape the upper and more distal intestinal resident microbiota composition,^81^, ^91^, ^106^ although the specific taxonomic changes are variable across laboratories.

The development of gnotobiotic models that re-conventionalize previously germ-free mice with fecal microbiota from malnourished children suggest that disruptions in resident microbiota are relevant for the pathogenesis of EED. Transfer of fecal microbes from children with kwashiorkor-type protein malnutrition into germ-free mice show that intestinal microbiota of malnourished children together with a nutrient-deficient diet recapitulate metabolic perturbations resembling kwashiorkor-type donors and concomitantly reduced growth recovery after food supplementation, even without other EED-like changes in the mucosa.^114^ Kwashiorkor-type malnutrition features, including hypoalbuminemia, edema, and increased circulating LPS, and villus blunting, also develop in calorie-restricted gnotobiotic piglets conventionalized with fecal microbiota even from healthy human infants. In gnotobiotic models, the most severe phenotypes have resulted from selective colonization with IgA-bound fecal microbiota, consisting of members of the enterobacteriaceae family, including *E coli* pathotypes, among other taxa.^115^ In addition to severe weight loss, these IgA-bound microbiota promoted crypt atrophy and epithelial cell discohesion, bacterial translocation, and mucosal immune activation.^115^ In contrast, fully nourished gnotobiotic piglets conventionalized with fecal microbiota from children with increased EED biomarkers did not develop histopathologic features of EED.^107^ In mice with moderate PM, the BG cocktail, but neither *E coli* nor the Bacteroidiales members alone, or other combinations of intestinal microbes, led to altered villus height:crypt depth ratios, increased permeability, and TJ aberrations. Thus, innovative models have established translational links between malnutrition/EED and dysbiotic microbial communities, although it has yet to be established whether in the absence of nutrient deficiency such altered microbiota alone are sufficient to recapitulate EED.

Dysbiotic microbiota and intestinal pathogens likely interact to influence EED. Despite no apparent direct intestinal pathology, the microbiota from children with EED allowed for more severe rotavirus diarrhea in gnotobiotic piglets.^107^ However, continuous depletion of resident microbiota with antibiotics prevented *Giardia*-mediated growth restriction, whereas *Giardia* modestly increased bacterial load in the upper small intestine during PM.^91^ A similar regimen of antibiotics given for 3 days before EAEC042, ETEC, or *Campylobacter* challenge markedly enhanced disease and allowed for up to approximately 3-log–greater peak shedding^97^ during zinc deficiency.^93^, ^97^, ^101^

### Disrupted Host-Microbial Cometabolism

EED in malnourished children is associated with perturbations in several host and intestinal microbial metabolic pathways. Serum changes are indicative of secondary carnitine deficiency, blocked fatty acid oxidation, dysregulation of sulfur amino acids (increased taurine and cystathionine), increased β-aminoisobutyric acid, and decreases in hippurate, ornithine, citrulline, and tryptophan.^116^, ^117^, ^118^ Conversely, greater levels of citrulline (in girls) or tryptophan (in boys) predicts better growth.^24^ Altered intestinal microbial metabolism is evident by increases in microbial-dependent exogenous breakdown of aromatic amino acids (ie, increased *N*-phenylacetylglycine and 4-hydroxyphenylacetate glutamine [from phenylalanine], cresol-sulfate [from tyrosine], and indole metabolites [from tryptophan]).^118^ The presence of these metabolites, however, may be dependent in some part on intestinal absorptive capacity.^73^ Increased trimethylamine oxide (TMAO) in stunted children is indicative of increased oxidation of microbial-mediated exogenous choline breakdown,^74^, ^79^, ^119^ and suggests potentially shared pathways with microbial influences on the metabolic syndrome.^120^

In animal models, PM alone leads to expected reduction in host catabolites of several amino acids (including phenylalanine, valine, leucine, lysine, and ornithine). There is also a shift toward carbohydrate metabolism seen as increases in TCA intermediates. Microbial-derived exogenous choline metabolites (methylamine, dimethylamine, TMA, and TMAO) also are increased. However, *N*-phenylacetylglycine, 4-hydroxyphenylacetate, and fatty acid oxidation intermediates are decreased. Enteropathogen challenges during PM show both pathogen-specific and shared metabolic profiles that compound the effects of PM alone. *C parvum*^119^ or EAEC infection^91^ further increase TMAO, whereas TMA and TMAO are transiently decreased after *Campylobacter* challenge,^101^ and *G lamblia* results in sustained decreases in TMA and exogenous choline and phosphatidylcholine breakdown.^91^ These metabolic influences of *Giardia* are sufficient to diminish TMA and TMAO increases in EAEC co-infected mice. In all cases studied, pathogens drive increased microbiota-dependent exogenous breakdown of tryptophan, phenylalanine, and tyrosine despite variable changes in their accompanying 16S ribosomal RNA profiles.^83^, ^91^

## Applications: Diagnostics and Preclinical Interventions to Remediate EED

### Biomarkers of EED

Given that the majority of children with EED show no symptoms, there is great interest in validating diagnostic biomarkers for this subclinical condition. Broad characterization of phenotypes and multi-omic profiling in animal models of EED and EED-like conditions may help identify common pathways that could be used as diagnostic markers, determinants of therapeutic response, or identifiers of at-risk children before development of stunting, cognitive decline, or metabolic syndrome.^18^ To date, markers of intestinal function/barrier loss (L:M ratios, fecal α1-antitrypsin [A1AT], fecal Reg-1, plasma zonulin), bacterial translocation (circulating LPS or anti-LPS antibodies), intestinal inflammation (fecal markers fMPO, LCN-2, lactoferrin, FC, and neopterin), systemic inflammation (serum markers CRP, serum amyloid A, and AGP), microbial exposures (molecular-based pathogen detections), and endocrine/metabolic markers (growth hormone and insulin-like growth factor 1 axis) all have provided insight into EED pathogenesis, but across multiple populations their interpretation requires complicated expertise in biostatistics and data integration.^64^ For example, among children followed up in longitudinal studies, stunting at enrollment was correlated with increased circulating LPS, but poor subsequent growth is better predicted by markers of diminished absorptive/epithelial cell function.^24^ In some settings, indicators of epithelial cell injury (A1AT and Reg-1 together with plasma zonulin) associate with poor growth only when combined with fMPO. fMPO alone correlates with systemic inflammatory markers (high-sensitivity C reactive protein, serum amyloid A, and soluble CD14).^24^ Across several studies (including Northeast Brazil,^24^ Tanzania,^23^ and Pakistan^62^), systemic inflammatory markers and/or serum antibodies to bacterial ligands (LPS and/or flagellin component FliC) were associated with impaired linear growth. Although systemic markers correlated with low IGF-1 and/or growth hormone resistance, systemic inflammation does not consistently coincide with markers of intestinal injury.^24^ Thus, EED may not operate entirely through systemic inflammatory pathways, desynchrony may exist between epithelial injury and resultant chronic intestinal/systemic inflammation,^25^ and/or confounding extraintestinal inflammatory drivers also contribute to poor childhood growth. Composite metrics incorporating several of these markers (ie, fMPO, neopterin, A1AT) may improve the predictability of growth impairment,^59^ but these scoring systems remain to be validated across multiple populations. In addition, age, sex, and dietary factors (including amount of intake from breastfeeding) can confound several of these assays.^60^, ^71^

In animal models, temporal relationships between a defined exposure to a putative EED trigger and/or nutrient deficiency and these or other biomarkers are being characterized, but mechanisms accounting for resulting growth impairment, such as persistent systemic inflammation even after intestinal restitution, have not yet been elucidated.

### Preclinical Therapeutics

Antimicrobial, nutritional, immune-mediated, and probiotic interventions in EED models can provide insights into potential novel therapeutics (Table 1). Nitazoxanide, a Pyruvate:ferredoxin oxidoreductase antagonist with anti-anaerobic and antiparasitic properties, can partially rescue EAEC042 infection during moderate PM, but nitazoxanide is ineffective at reducing the severity of cryptosporidiosis.^92^, ^94^ Amixicile, however, a water-soluble nitazoxanide derivative that lacks anticryptosporidial activity, can benefit the growth of mice with PM even after *C parvum* challenge.^121^ Depletion of resident microbiota has differential effects depending on the specific pathogen challenge. Oral replenishment of specific amino acids (such as alanyl-glutamine) can support IEC proliferation and partially restore host growth,^80^ even during *Cryptosporidium* challenge.^94^ Interestingly, the full benefit of alanyl-glutamine may depend on combining treatment with targeted antibiotics and/or modulators to reduce overexuberant host inflammation.^122^, ^123^ Parenteral delivery of arginine also can attenuate the intensity of *Cryptosporidium* infection and the severity of growth impairment in undernourished mice through pathways of both defense-promoting induction of nitric oxide as well as arginase pathways important for IEC restitution.^124^ Tryptophan increased systemic and mucosal T-regulatory cell numbers in both nutrient-deficient and -sufficient piglets,^125^ however, tryptophan may worsen certain infections,^126^ including *Cryptosporidium* during protein malnutrition (D.T.B. and R.L.G., unpublished data). Intestinal microbiota from healthy donors can strikingly restore growth and metabolic function during multinutrient malnutrition,^127^ and even some specific taxa (ie, *Lactobacillus plantarum*^*WJL*^)^128^ may be sufficient to promote growth despite diminished nutritional intake. Co-colonization with *Akkermansia mucinophila* and *C scindens* isolated from stools of healthy child controls was able to mitigate the deleterious effects of the IgA-bound enterobacteriaceae.^115^

### Emerging Models and Reductionist Systems

In addition to murine models, advances in human organoid systems for the study of intestinal pathogens is rapidly expanding.^129^, ^130^, ^131^ Extension of findings in murine models into these and other systems (ie, humanized mice, human-derived enteroids, organoids, and gut/organ-on-a-chip technologies) will be critical for validation and to overcome notable limitations in translating animal models to human disease. Such models also can represent powerful new tools to elucidate pathways by which EED therapies may restore gut healing.^132^

## Conclusions

It is clear that our understanding of the complexities driving EED remains superficial at best. Comparisons and contrasts between findings in these early EED models and ongoing studies in child cohorts suggest that EED may be a heterogenous condition, resulting from a convergence of dynamic microbial and nutritional factors that may be either episodic or persistent, and result in lingering disruptions even after the primary insult has cleared. Until these diseases other than overt diarrhea are named, they remain inadequately counted and are overlooked in key analyses of the impact or of the effectiveness of preventive or therapeutic interventions. Therefore, we have suggested 3 respective names for 3 major EED disease outcomes as: HAZdrop (for the reductions in HAZ scores in the first 2 years of life), COG-hit (for the cognitive impairment hit of normal cognitive development attributable to early childhood enteropathy), and MET-syn (for the later-life metabolic syndrome that is being appreciated as increased in those who had experienced early childhood enteric infections.^14^, ^17^ As sequelae of EED beyond growth restriction emerge, applying EED models to identify specific microbe–microbe and microbe–host interactions that can sustain mucosal health and defense, promote intestinal restitution, preserve physical and cognitive development, and avert metabolic syndrome becomes increasingly important for improving the health of children around the globe.

## Supplementary Material

Supplementary Table 1 Models of EED and EED-Like Conditions as They Relate to Descriptions of EED in Children
